# How Does Railway Respond to the Spread of COVID-19? Countermeasure Analysis and Evaluation Around the World

**DOI:** 10.1007/s40864-021-00140-z

**Published:** 2021-03-04

**Authors:** Yonghao Yin, Dewei Li, Songliang Zhang, Lifu Wu

**Affiliations:** 1 Institute of Artificial Intelligence and Robotics (IAIR), Key Laboratory of Traffic Safety on Track of Ministry of Education, School of Traffic and Transportation Engineering, Central South University, Changsha, 410075 Hunan China; 2 State Key Lab of Rail Traffic Control and Safety, School of Traffic and Transportation, Beijing Jiaotong University, Beijing, 100044 China

**Keywords:** COVID-19 transmission, Railway response, Countermeasure evaluation, Public health

## Abstract

The global COVID-19 pandemic is having a significant impact on the development of many aspects all over the world. As an important part of public services, rail transit requires effective response countermeasures to control the spread of COVID-19. Considering the current development of the epidemic situation, this article discusses the characteristics of COVID-19 transmission and identifies vulnerable areas to target in order to prevent and control the spread of the epidemic in the rail transit system. Countermeasures adopted to prevent the spread of COVID-19 are analyzed in terms of external and internal categories, which were classified into six groups: passenger service, case care, information, staff, equipment and operation management. An evaluation architecture was also constructed, which was established from the perspective of effectiveness, economic efficiency, acceptability, privacy and so on. The effect of implementing the measures was evaluated by a social survey, and their advantages and shortcomings were analyzed, which can be used to guide future epidemic prevention and control for rail transit systems around the world. It is important to formulate a reasonable work schedule according to local conditions, providing a reference for rapid response to future public health emergencies of international concern.

## Introduction

**Fig. 1:**
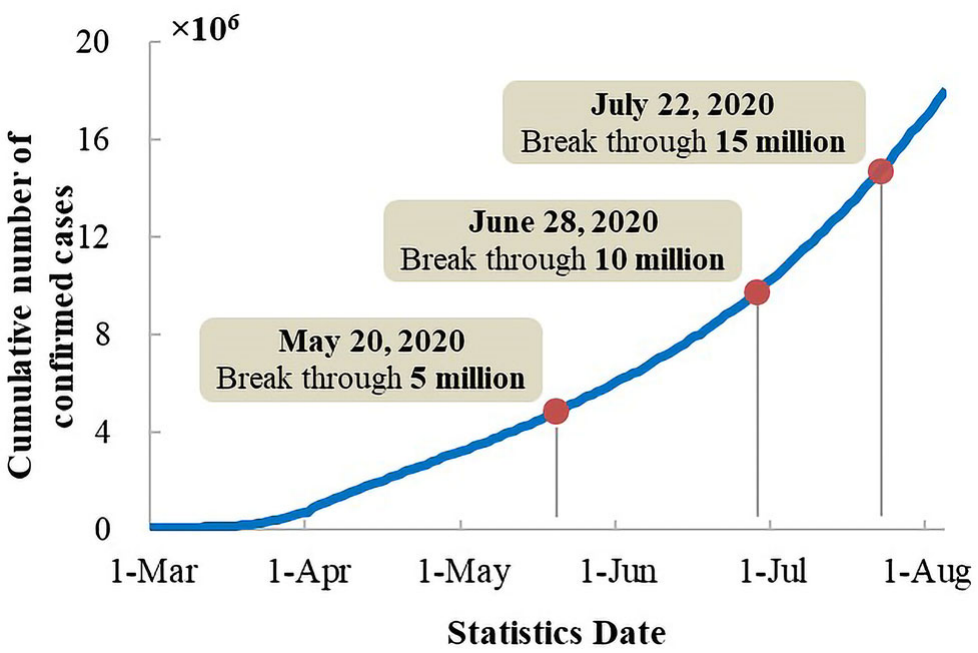
Changes in cumulative number of confirmed cases of COVID-19 globally

With the outbreak and continuous spread of COVID-19, as of July 13, 2020, a total of over 13,000,000 COVID-19 cases have been confirmed globally, with a cumulative number of deaths of over 560,000. Figure 1 shows the cumulative confirmed cases of COVID-19 around the world since March 1, 2020, as of July 26, 2020. The World Health Organization (WHO) declared COVID-19 a global pandemic, considering that the epidemic cannot be eliminated in a short time, and small-scale interpersonal transmission of the virus may appear all over the world. At the same time, the virus is always at risk of mutation. How to deal with such a global public health emergency has become a major global challenge that needs to be addressed by all countries around the world.

After the outbreak of COVID-19, a lot of countries around the world took urgent measures to cut off the transmission of the virus. People gathering, movement and transfer may cause the spread of the virus from person to person, so it is necessary to close or control public places and reduce the mobility strictly. The traffic and transportation industries are responsible for the emergency transport of materials for epidemic prevention and control. On the other hand, such mobility can lead to the spread of COVID-19 through transfer of the virus among a large number of passengers in closed vehicles within a short period of time. As the railway is a high-density traffic mode, it is a great challenge to control the spread of the virus via rail traffic while maintaining a basic level of service, let alone guaranteeing the operational profit of railway companies. With the continuous increase in travel speed, the spread of the virus accelerates by rail transit. Although many countries have tried various countermeasures, from encouraging passengers to wear masks to reducing gathering of passengers on the platform, the question as to “what kind of countermeasures should be taken during the pandemic” remains open.

Despite very limited research on preventing the spread of COVID-19, some research on other virus pandemic can be found. The International Ebola Response Team et al. (2016) presented analysis of data collected during the Ebola epidemic in West Africa and highlighted parts where control could be improved [1]. Kramer et al. (2016) predicted the spread of the Ebola epidemic through assessment [2]. Due to the characteristics of dense traffic and closed spaces, the range of transmission of the virus can reach 4.5 m in public transport, and it is more likely to have cluster cases in public transport. As a result, in the field of transportation, how to control the spread of the virus is also an important topic. Konstantinos et al. (2020) identified some intervention measures that can support public transport [3]. Colizza et al. (2006) studied the role of air transport networks in determining the global spread patterns of emerging disease [4]. Zhao et al. (2020) discussed the relationship between train transportation and the spread of COVID-19 in a data-driven way [5]. Alejandro et al. (2020) outlined the measures to reduce crowding in public transportation [6]. Zheng et al. (2020) examined correlations of daily frequencies of each transportation method and the distance between Wuhan and other cities, with the daily number and the cumulative number of COVID-19 cases. They emphasized that strong preventive measures should be taken in cities with shorter distances and more frequent public transportation connectivity with the epicenter to contain the COVID-19 epidemic [7]. Chu et al. (2020) investigated the effects of physical isolation and the use of masks and goggles on the spread of the virus [8]. Hu et al. (2020) discussed the risk of COVID-19 transmission in train passengers. They found that the transmission risk is heterogeneous depending on co-travel time and seat location, and the passengers adjacent to the patient have the highest risk [9]. Therefore, measures should be taken to prevent virus transmission in the rail transit system.

As a part of public transportation, how to prevent the spread of COVID-19 in rail transit systems is also a key concern of government transportation departments. Due to the different national conditions such as the culture, economy and social education level, the traffic response to the epidemic also varies. Practice shows that the isolation of infectious sources, active treatment, public cooperation and other methods may effectively control the spread of the epidemic. On the other hand, some countries are worrying about the possibility of railway companies becoming bankrupt and the potential negative influence on economy and society. Given that many countries are still in the outbreak phase, it is important for different countries to share their experiences in the global response to the outbreak, the full resumption of production and the future development of epidemic prevention plans.

The objective of this study is to review and assess the current countermeasures been taken in the railway industry around the world. This paper summarizes the countermeasures taken by the railway operators from various countries to deal with the outbreak of COVID-19 from the aspects of external management and internal management. External management includes information dissemination, passenger service and suspected case handling, while internal management includes employee management, equipment management and operation management. Finally, the evaluation indicators are established to respond to the epidemic and evaluated to compare their advantages and disadvantages, which provides suggestions for countries to better fight the epidemic and respond more quickly to public safety emergencies in the future.

## Spread of COVID-19 Through Rail Transit Systems

To introduce effective policies for the railway industry, it is necessary to discuss the transmission mechanism of the virus and the operational characteristics of railways. Afterwards, the relationship between virus transmission and rail transit system operation can be investigated and the vulnerable spots can be highlighted.

### The Transmission Characteristic of COVID-19

COVID-19 is caused by the virus named SARS-CoV-2. Chen (2020) found that the SARS-CoV-2 is not stronger than SARS, either from the fatality rate or R_0_ (R_0_ implies the basic reproduction number in epidemiology, referring to the average number of people infected with an infectious disease by a person who is infected with an infectious disease without external intervention under the circumstance that no one is immune; the larger the R_0_ number, the harder it is to control an epidemic) [10]. However, it is more infectious than the Ebola virus and Middle East Respiratory Syndrome (MERS), which means that there are more confirmed cases of SARS-CoV-2. Table 1 shows the fatality rate and R_0_ of common viruses all over the world. Therefore, there is a high probability that the virus will spread around the world. If the prevention measures are negligent, local outbreaks may take place.

**Table 1:** Common viruses and corresponding fatality rate and R_0_

Virus name	Fatality rate (%)	R_0_
SARS-CoV-2	3	1.4–5.5
SARS	10	2–5
MERS	40	< 1
Measles virus	0.3	12–18
Ebola virus	70	2.3
HIV	80	3.4

A cluster outbreak of COVID-19 is determined based on whether the confirmed cases are found in the family, shopping malls, parties, construction sites or other places. It is also defined when more than two cases are diagnosed as COVID-19 infection pneumonia or suspected cases due to close contact within 14 days. Experimental results from Chen (2020) have shown that the COVID-19 virus can survive for up to 72 hours on plastic and stainless steel, less than 4 hours on copper and less than 24 hours on cardboard [10]. In environments with higher humidity aerosols, COVID-19 virus can also survive for more than 3 hours. As a result, once the virus appears in crowded and fluid closed environments (e.g. train carriages, underground platforms and stations, restaurants and other confined public spaces), its survival time is long enough to infect thousands of people.

Moreover, the main transmission routes of COVID-19 are droplet spread, contact transmission and aerosol propagation.

*Droplet spread *The disease spreads primarily from person to person through small droplets from nose or mouth, which are expelled when a person with COVID-19 coughs, sneezes or speaks. People can catch COVID-19 if they breathe in these droplets from a person infected with the virus.

*Contact transmission* Droplets can land on objects and surfaces around the person, such as tables, doorknobs and handrails. People can become infected by touching these objects or surfaces, then touching their eyes, nose or mouth. This is why it is important to wash hands regularly with soap and water or clean with alcohol-based hand rub.

*Aerosol propagation* If the infected person is in a confined space, the virus is very easy to aerosolize and form biological aerosols, resulting in the virus surviving and being transmitted through the aerosol [11].

In summary, given the characteristic high spread rate of the virus and the various transmission routes, hospitals, offices, service areas, railway stations, trains, ships and other public places are the important areas which should receive more attention to prevent clustered outbreaks.

### COVID-19 Transmission in Rail Transit Systems

As one of the most important public services in each city, the rail transit system provides citizens their daily necessary travel both within and between cities. However, the closed carriages, stations and underground platforms are confined spaces, and travelers gather during peak hours, which may lead to clustered outbreaks of the virus. The main threats including external and internal threats are discussed before introducing the countermeasures.

1. External threats

*Input source* As one of the most crowded public places, the rail transit system is visited more than a million times per day by passengers. Therefore, the input source of the system should be strictly controlled to prevent the spread of the epidemic, which is one of the most vulnerable spots of the system. Specifically, passengers should be strictly screened before they enter the station, such as by taking temperatures, asking about the travel track and so on. Meanwhile, passengers are also asked to equip themselves with personal protection, such as wearing masks, washing hands frequently and so on.

*Gathering of passengers* Passengers will arrive at busy stations frequently and intensively in a short period of time during peak hours, which may lead to the gathering of passengers because of the process of security checking, card swiping and passage walking. Meanwhile, passengers may gather together when they have meals, do some shopping at stores, rest at the waiting room or board the train. In addition, the passenger density is quite large in crowed carriages. Droplet transmission and contact transmission will become the main means of virus transmission in rail transit systems when a large number of passengers gather with possible cases. The distance between passengers is sufficiently close for the virus to spread widely. Therefore, the passage, hall, platform and carriage should also be considered when attempting to maintain a safe social distance between passengers, which may effectively decrease the risk of disease spread. Controlling passengers with a safe social distance and setting reasonable train seat distances are the main measures to deal with external hazards at stations and aboard trains.

(2) Internal threats

*Working space* Since the staff are working in the same environment as high-traffic passengers, even if the staff are avoiding close contact with passengers, the virus may spread more rapidly among employees. As a result, measures such as strengthening staff protection, decentralizing staff distribution and restricting entry for regions are also necessary.

*Closed environment* As a long and narrow closed container, the rail transit vehicle is isolated from the outside, which changes the air only through the ventilating system. In the closed places of rail transit trains, saliva and other respiratory droplets can easily spread from patients to healthy person. Therefore, the railway ventilation system is the key facility to prevent the spread of the virus. Improving ventilation systems, keeping the sewer clear and paying attention to environmental disinfection are important means to prevent the outbreak and expansion.

*Operation plan* According to preliminary statistics, during the outbreak in China, from January 23 to February 10, 2020, the average daily passenger traffic in Chinese urban rail transit (except eight cities that were all suspended) decreased by about 70–90% compared with the same period in 2019. On February 10, after some enterprises resumed work, the average daily passenger traffic in urban rail transit began to experience a rebound. Among the increases, passenger traffic of Beijing, Shanghai, and Guangzhou increased by about 72%, 99%, and 48%, respectively, according to the resumption of work [12]. With the gradual resumption of work and production in many provinces and cities, the potential growth pressure of passenger flow is enormous. It is also a great challenge to formulate suitable operation plans, optimizing transportation processes and cooperating closely with the outside world.

## Countermeasures Adopted to Prevent the Spread of COVID-19

A series of countermeasures need to be taken in rail transit system to reduce the virus infection risks of public transportation and to control the spread of COVID-19 because of the limited airflow of the system and the dangerousness of the infectious diseases. As an external service department, it should adjust the train service, focus on the management of internal staff and inform users of the common sense of epidemic prevention.

As the worldwide professional association representing the railway sector, the International Union of Railways (UIC) published two questionnaires in March 2020 to survey the countermeasures adopted to prevent the spread of COVID-19 in the rail transit system. Fifty-seven UIC member organizations from 35 countries around the world participated in that survey. A database, the *UIC Covid19 TF_Data Base*, was proposed by UIC, which recorded the countermeasures in those countries to prevent the spread of COVID-19 in rail transit systems [13].

**Fig. 2:**
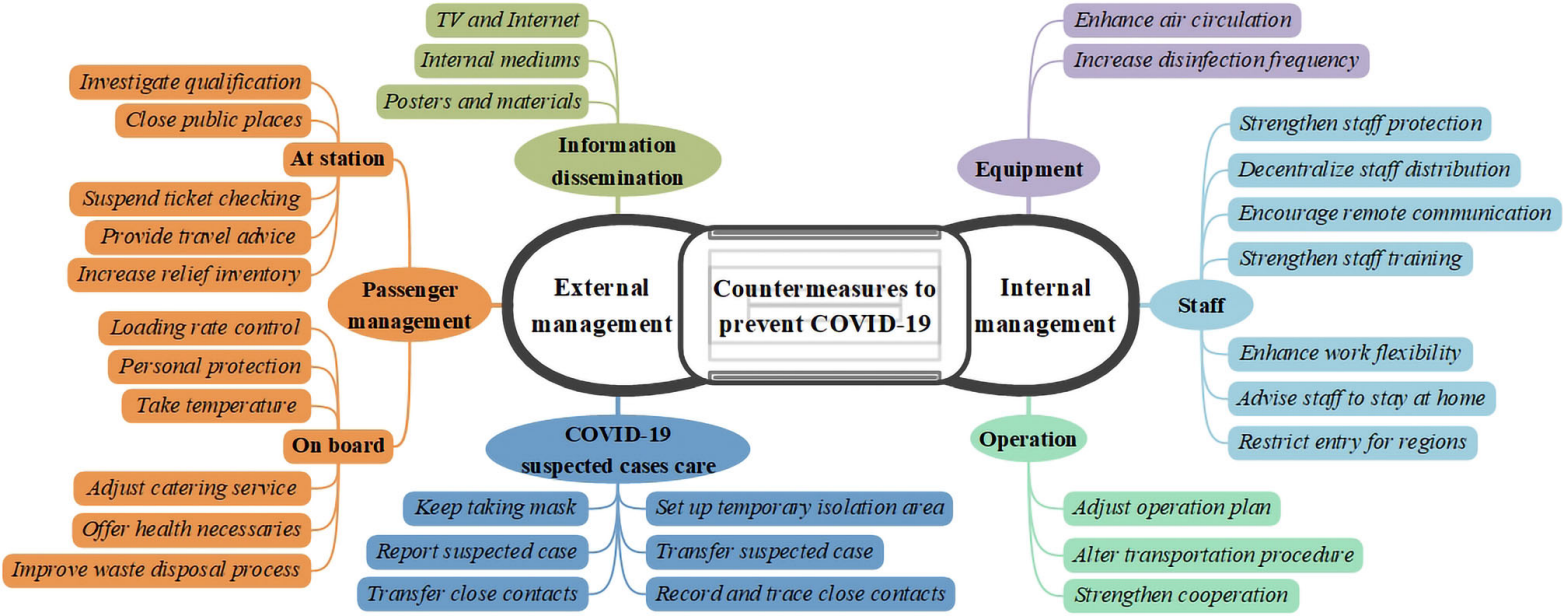
Outline of initiatives to prevent the spread of COVID-19 for rail transit

Based on the UIC survey, the countermeasures of rail transit systems in different countries around the world were collected and collated, and an outline of the epidemic prevention initiatives is shown in Fig. 2. The countermeasures are classified into two categories, six aspects and 32 items. Specifically, from a management perspective, the measures are divided into external and internal management countermeasures, and every category has three aspects, which will be discussed in detail as follows.

### External Epidemic Prevention and Control for Rail Systems

Rail transit systems provide the basic guarantee for mobility of society, and it is necessary to protect passenger lives and health. The spread of the virus should be controlled in the process of organizing passengers, and detailed measures should be developed to deal with suspected cases. In addition, due to the public attribute of rail transit, media resources are accompanied by it, which can be considered as a window for information sharing. Therefore, the external management countermeasures include three aspects: passenger management, suspected COVID-19 case management and information dissemination.

#### Passenger Management

In the rail transit system, passengers need to go through a series of processes such as entering the station, waiting for the train, boarding the train, exiting the train and exiting the station. In some countries, passengers should be checked at a security passage before they enter a station. If effective measures are not taken in the process, the moving passengers, crowded platforms and closed carriages are very likely to cause the spread of the epidemic. As the train is movable equipment and the station is a fixed place, the epidemic prevention and control measures should be adapted to different environments. Therefore, the passenger control and service measures on board and at stations will be analyzed independently in this section.

1. On board

The train is a moving device, which is a closed environment during operation, so the coronavirus will spread quickly in the limited space once it invades. In addition, passengers need to stay in the carriage for a long time during travel, which may greatly increase the duration of exposure to the virus and the risk of infection. Therefore, some necessary preventive measures should be taken on board.

*Loading rate control* To encourage passengers to maintain a safe social distance, controlling the loading rate of trains or seat occupancy rate are widely used approaches in many countries, such as in China, Canada, France, South Korea and so on. Because the primary way that COVID-19 spreads is by close person-to-person contact via droplets or skin touch, keeping a safe social distance (approximately 6 feet or 2 m [14]) can prevent the spread of the virus. For example, standing tickets are no longer sold in China during the outbreak. The train occupancy rate is limited at around 33% in Spain, 35% in Canada and 50% in China. Passengers are not seated directly beside one another, with passengers seated in just one seat out of two in France and South Korea. In Italy, some new rules for seat reservations are set, which ensure a minimum distance of more than 1 m.

*Personal protection* The onboard passengers are required to take personal protection measures such as wearing masks, washing hands, limiting close contact and covering their mouth and nose when coughing or sneezing. Washing hands frequently is one of the cheapest and most important ways to prevent the spread of the virus. Regularly and thoroughly cleaning one's hands with an alcohol-based hand rub or with soap and water can kill viruses that may be on passengers' hands. The process of washing hands is important but easily neglected. Specifically, to effectively eliminate the virus on passengers' hands, they should be washed for at least 20–30 seconds and follow a scientifically approved procedure. Furthermore, the time of washing hands should also be considered, especially after blowing one's nose, coughing or sneezing, after visiting a public space, after touching surfaces outside of the home, before, during and after caring for a sick person and before and after eating [15].

*Take temperature* Because one of the most typical symptoms of COVID-19 is fever, it is necessary for passengers to be screened. Considering that some passengers may pass the inspection by physical cooling before boarding, onboard passengers will be randomly selected for temperature checks to promptly identify suspected infection. Any passenger with body temperature of 37.3 °C or above will be immediately isolated and then transferred at the next station for medical check.

*Adjust catering service* The catering service is adjusted or even canceled on the train. It is necessary to control the catering service to prevent the spread of the virus because passengers will take off their masks and eat. As a result, the use of dining cars is advocated in some train systems. It is suggested that operators deliver all foodstuffs and beverages to passengers as a package and covered. Furthermore, all the glasses, plates and cutlery should be single-use and disposable.

*Offer health necessities* The operators provide protective equipment such as masks and bottled disinfectant for passengers to prevent viral infection and transmission. To implement this policy, the crews provide free masks for passengers, if they lost their masks at the station or on board, and the passengers are also asked to take masks before they enter the station. In addition, the crews are asked to avoid physical contact and to not touch passengers’ personal belongings whenever possible. The crews should also carry a disinfectant whenever possible, and managers are requested to arrange for disinfectants to be provided in their area of responsibility.

*Improve waste disposal process* The used masks, tissues and other trash may carry a lot of viruses, so the disposal of the special trash properly is important. Waste may cause biochemical pollution, especially for masks and used tissue, so they should be put into a bag and then thrown away in the trash. Bags have been put in all compartments for travelers' hygienic rubbish. The bags are tightly closed and placed in the designated places when they start to be used.

(2) At station

To keep the passenger crowd level of the rail transit system within an acceptable range to prevent spread, it is necessary to take measures not only on board but also at stations to guarantee the service level for passengers entering the system. Meanwhile, the management of the station hall also cannot be ignored, which can cause crowds to gather. The specific measures are as follows.

*Investigate qualification* Pre-boarding temperature screening measures can be performed to avoid suspected patients' access to public spaces. Infrared automatic thermometers can be installed at major stations, and all passengers’ temperatures are taken at the station entrance or exit. Additionally, to protect the safety of passengers and staff, the suspected patients are controlled and asked not to board the train. This measure can ensure that most of the passengers of rail transit service are all healthy, which is efficient to control the virus spread at the source, even if it may be troublesome for some passengers. Specifically, in China, a health quick-response code (health QR code) has been proposed by the government, which can reflect the health condition of a person. The citizens apply for the health QR code on a mobile-phone application, and the health condition will be updated all around the country. If the citizens are patients or have made contact with suspected patients or stopped at a high-risk region, the health QR code will reflect the information with a red color; if citizens are suspected of making contact with patients or stopping at a medium-risk region, the health QR code is yellow; otherwise, it is green. The passengers' health QR code will be checked when they enter the station, and only the passengers with a green code can get through. The passengers with other color codes are asked to stay at home and reduce unnecessary trips.

*Close public places* Just like the restaurant on board being closed, operators have closed public places, such as restaurants, shops and passenger waiting rooms of the stations, which can avoid the indirect contact with others and reduce passenger gathering.

*Suspend ticket checking *In some railway systems, ticket checking is mandatory before passengers can gain access to the platform. During the pandemic, the staff welcoming and ticket checking at station exits can be suspended or canceled to avoid unnecessary gathering of people and avoid contact to protect staff.

*Provide travel advice* Operators provide some travel tips to passengers, which can help reduce the risk of viral infections. For example, they ask passengers to wear masks, avoid walking into crowds of people, comply with minimum interpersonal distance and to not eat food at stations.

*Increase relief inventory* Since disinfection and cleaning have become daily routine tasks, the necessary material reserves are indispensable. The government should specifically introduce policies to guarantee the supply of disinfection and monitoring equipment and increase the stock of antiviral drugs. The mentioned materials include hand sanitizer, disinfectant, protective clothing and spare medicine. In addition, a certain number of masks should be prepared for passengers if their mask falls or breaks.

#### Suspected COVID-19 Case Care

Suspected COVID-19 cases generally have symptoms without nucleic acid testing, such as cough, fever, shortness of breath or fatigue. If the diagnosis is confirmed after testing, they will be converted to a confirmed case. Passengers in this situation may also be contagious due to the presence of viruses. To minimize the risk of viral spread, proper treatment of the suspected cases is required. The process of taking care of suspected cases in the railway is very important and necessary in epidemic prevention and control. Based on the survey of the affected countries, a series of successive measures is summarized below.

Just like the management of passengers, the handling of suspected cases can also be divided into two circumstances: on board and at the station. The circumstances of the two scenarios are different, so the corresponding measures can also be diverse. Generally, the stations are in an urban region and have seamless connection with medical departments. As a result, measures for taking care of the suspected COVID-19 cases can be easier at stations. Meanwhile, the treatments on board may be complicated, but the main processes are similar. The main processes of handling the suspected cases include keeping the suspected cases wearing masks, setting up a temporary isolation area, transferring them through the specialized access, transferring the close contacts to a safe place and observing them regularly, reporting the information to the related units, and recording and tracing close contacts. The specific measures of the above process are listed sequentially as follows.

*Keep suspected cases taking masks* To reduce the risk of infection for other passengers, the suspected cases are asked to keep wearing a mask. If the mask of the sick person is lost, they will be given an unopened mask so they can take the mask themselves and use the mask following the instructions in the information sheet.

*Set up temporary isolation area* A temporary isolation area should be set up at a specific space of the train or the station. When the suspected cases are discovered by taking temperature, they will stay in the temporary isolation area until they are transferred.

*Report suspected case*s Information on the suspected cases will be relayed to the related units (such as the dispatching office or the centers for disease control) by the known reporting channel. The transfer measure and necessary coordination will be scheduled.

*Transfer suspected cases *The suspected cases should be transferred to the temporary isolation area at the first time, which minimizes contact with healthy people. Then, based on the communication with the related units and the transfer plan, the passengers with a fever will be timely delivered to the scheduled station with a health check-up station. They will be transferred to the local epidemic prevention department for further medical checks and treatment.

*Transfer close contacts *The passengers in the sick passenger’s immediate vicinity will be asked to move to a safe place and be observed regularly. Wherever possible, people are prevented from entering and leaving the area where the sick passenger is located. Meanwhile, the operator will provide guidelines for managing the suspected and infected individuals.

*Record and trace close contacts *All people (including train crew) who came within 2 m of the sick passenger will be given forms (including identity, contact information) and asked to fill them out. After screening, if the fevered passengers are either confirmed or suspected patients, the operators will immediately inform the local epidemic prevention department and report information of their close contacts, to make it easy for authorities to trace them. Infected persons should be traced as part of the infection tracking process as clearly as possible. Meanwhile, the confirmed cases’ information will be published and announced to the other passengers on the train. In addition, considering the mobility of people, this information should also be reported to the national unified information platform, in order to meet the need for big data search later.

#### Information Dissemination

As a part of the public service system, the information system of rail transit (such as broadcasting, screening, signs, etc.) is one of the most important media to disseminate information. When an outbreak occurs, the rail transit system plays a significant role in providing travel and epidemic prevention information for passengers and citizens, as passengers may not understand the different operation control countermeasures with the normal situation.

Specifically, information dissemination measures can be classified into online measures and offline measures. The online measures include information dissemination by TV, official websites, mobile apps, social networks and e-mail. The offline measures include information dissemination through public announcement systems and staff.

*Release information using TV and the Internet* Operators have continuously and timely updated the information related to the epidemic outbreak, updated prevention measures and organizational measures through online media tools such as TV, official websites, social networks, mobile apps and e-mail. Meanwhile, they have disseminated knowledge on the virus prevention and information regarding coronavirus to passengers, which includes the cause of the disease, symptoms of the disease, ways of transmission, control and prevention, etc.

*Disseminate by internal mediums* Operators have played continuous looped promotional videos and announcements of prevention measures with different languages through public announcement systems such as surveillance service, information displayer and broadcast at stations and onboard. Besides, some staff were briefed on proper hygiene measures to support passengers.

*Provide posters and materials* Operators have published and provided leaflets, brochures and initiative materials to travelers to raise awareness among passengers regarding symptoms and hygiene practices, including information about symptoms, personal protective measures and social distancing measures. In addition, posters about prevention information have been published at bulletin boards, and prevention measure posters have been displayed on bathroom doors, mirrors, ticket offices and business lounges.

### Internal Epidemic Management for Rail Systems

In addition to the prevention and control for passengers in the rail transit system, it is also very important to ensure the safety of the staff. This is essential for the normal operation of the system, because railway staff connect with hundreds or even thousands of passengers. One staff case could cause severe spread of the virus in the network. The virus may remain on the surface of the equipment which the virus carriers touched. Therefore, the equipment in the rail transit system that includes trains and station communal facilities should be timely disinfected during the epidemic period. Moreover, the normal operation rules may also be broken, which need to be promptly adjusted to accommodate passengers' demands during the specific period. The internal measures to ensure the normal operation of the rail transit system include the following aspects: staff management, equipment management and operation management, which will be discussed in detail as follows.

#### Staff Management

The staff can be regarded as a virus-susceptible population, because they contact passengers frequently every day, especially the trainmen and ticket sellers. Therefore, measures for passengers are also applied for the staff, such as taking masks, taking a temperature before working, keeping safe social distance and so on. Meanwhile, in allusion to the processes of shifting work, meeting and training, some special measures need to be taken to prevent and control spread during the epidemic period.

These general protection measures are being taken by most countries, such as wearing masks, taking body temperature before working, reducing the distribution density of staff in the workplace, prohibiting fever people from taking jobs and distributing protective equipment for employees. In allusion to the processes such as shifting work, meeting and training, some special measures need to be taken to prevent and control the spread during the epidemic period as follows.

*Strengthen staff protection *To ensure the normal operation of the rail transit system, the health and safety of staff should be the first priority. As general protection measures mentioned above, to strengthen staff protection, additionally, if the face masks are disposable, they should be replaced every 6–8 hours for staff on long duty [16].

*Decentralize staff distribution* It will greatly prevent contact between people because physical isolation can effectively cut off the path of the viral spread. Considering the closure property of workplaces, once an employee is infected with the virus, it will spread quickly in a closed space. Some measures should be taken into consideration to insulate the internal official area from the external environment and decentralize the distribution of the staff. For example, rail transit departments may set up separate passages, elevators and toilets for employees to avoid sharing the same facility with passengers. To reduce the shift frequency, the staff can be rearranged to change the working shift system. The staff who may come into contact with the passengers are organized and managed independently, which can prevent internal staff contact with external people.

*Encourage remote communication* Assembly can easily cause people to become infected, which often occurs in public places such as hospitals. To ensure the safety of staff, scenes where many people gather should be avoided as much as possible. Without face-to-face meetings, they can take the form of online office, which not only reduces the contact, but also makes the time arrangement more flexible. The companies encourage people to use the Internet and communicate by email. In addition, the private network is also the main medium for transmitting information and by which some companies have decided to release information.

*Strengthen staff training* Training staff to achieve the knowledge and measures of preventing virus spread can improve the awareness of staff to prevent the virus. They should ensure their own health firstly before they serve others. As a working skill, the measures of preventing virus spread in rail transit system should be available for every employee. There are many countries taking action to train staff, but the content of training in different countries is slightly different. Specifically, it mainly includes the following four specific contents. First of all, let the staff master the management procedures for suspected cases and confirmed cases, which is a basic ability that the staff should have. Furthermore, it is important for staff to assess their health status. If they suspect that they have symptoms, they should be immediately quarantined at home in time to avoid bringing the virus into the workplace. In addition, they should have sufficient knowledge of COVID-19 prevention and control, not only to protect themselves, but also to improve the ability to identify infected people. Some countries have adopted training to encourage staff to pay attention to work etiquette and personal hygiene.

*Enhance work flexibility* In response to the special situation during the epidemic, employees may also encounter various difficulties correspondingly. To enhance the care for them and allow them to work on the job better at the same time, it is necessary to adjust the flexibility of daily work. Flexible work systems could be attempted, which provides employees with a certain degree of freedom. Some companies minimize the number of workers in positions exposed to the outside world and strive for the health and well-being of employees. For employees who are in poor physical condition and have low immunity, a special holiday is a favorable measure to provide employees with health protection. Due to school closure in some places, many staff’s children are left unattended. Therefore, some companies have specifically allowed employees to bring their children to work and set up a special place for guardianship.

*Advise staff to stay at home in spare time* In addition to regulating employees' behavior in the work process, if precautions of daily life are neglected, the virus will also be carried into the working place. As a public service industry, the health condition of the staff in the system should also be strictly guarded during non-working times. Prohibiting employees from going to high-risk regions can greatly reduce the possibility of infection. In order to ascertain the employees' disease history, more precise measures can be promoted gradually, such as prohibiting employees from transiting at local airports in high-risk regions. The employees are advised to avoid going to the hospital or other crowded places. Last but not least, quarantining employees who travel to high-risk regions is also an effective means.

*Restrict entry for some working regions* Reducing the movement of people is the most traditional measure for epidemic prevention and control. Some places such as control rooms and ticket offices do not need to be open for all staff, and the number of people who enter these areas should be strictly limited. The operators in Japan and Belgium have set up some areas where unrelated people are not allowed to enter, and the channels of employees have been optimized to reduce the possible spread of the virus.

#### Equipment Management

Facilities and equipment such as passenger station halls, platforms and trains ensure the daily operation of public places, but they will also become a primary way for the virus to remain and spread while in contact with different people. In order to cope with the epidemic situation, the use of equipment should also be adjusted accordingly for changes.

*Enhance air circulation* The ventilation equipment inside the train has been reformed, the main purpose of which is to enhance the air circulation. The same situation also happened on the ventilation equipment of the station. As a respiratory disease, COVID-19 can spread through droplets. In a closed space, the virus will show greater lethality, when it reaches a certain concentration. Frequent ventilation is mainly to reduce the virus concentration. When fresh air circulates and the droplets carrying the virus fall to the ground, the virus' pathogenicity will decrease, and the chance of infecting others will be less.

*Increase disinfection frequency* Basically, each country all over the world regularly disinfects every kind of equipment in the rail transit system. From fixed equipment to mobile equipment and from the surface of the object to the whole equipment, it is necessary to arrange for dedicated personnel to perform virus killing and cleaning work. The disinfection operation mainly includes two levels, by fixed cyclical time and in specific location. The common disinfection areas at stations include security equipment, gate machines, elevator cars, seats, toilets, handrails and recycled ticket cards. The train should focus on the columns, armrests, seats, doors and other parts which are easily accessible by passengers. Apart from that, air-conditioning filters should also be replaced regularly. The depot should also be thoroughly disinfected in the high-risk regions. The stations and vehicles should be disinfected and cleaned at different frequencies according to the level of region risk. In China, for example, the key equipment in the station that passengers use frequently, such as toilets, ticket gates and escalators, are disinfected every 2 hours for the high-risk regions (such as Beijing in China in June 2020); the hall and platform of stations in low-risk regions (such as Changsha in China in February 2020) are disinfected two times per day. In addition, except for the planned disinfection operations, specific disinfection in response to suspected cases care should also be considered. The space that suspected cases touched and remained in should be promptly disinfected. Meanwhile, the carriage where suspected cases stayed should be disinfected on time. As soon as the train arrives at the car depot, the whole train also should be comprehensively disinfected.

#### Operation Management

The occurrence of COVID-19 has led to variation in the relationship between supply and demand. To meet the evolution of passenger travel, the operation service should be adjusted in a timely manner. The specific performances are as follows.

*Adjust operation plan* The epidemic has drastically changed passenger travel demand. At the same time, it is necessary to consider the density and evacuation of personnel while transporting passengers. Especially for some countries to which international passenger travel frequently, the supply structure of the transportation system should be adjusted accordingly. In view of this situation, the external border services of rail transit ought to be shut down, and the train stopping at some specific cities (high-risk regions) should be directly canceled. Meanwhile, passengers are informed about the risk of traveling and advised to travel during off-peak hours instead of crowding during peak hours. Reducing the frequency of train operation also proves to be a useful method. In some countries, such as Japan and Spain, the frequency of trains is decreased to control the number of travel passengers during the outbreak. On the contrary, the train frequency is increased to reduce the passenger loading rate of each carriage in some countries (such as China) during the resumption stage of working and production, which not only can guarantee the daily trips of citizens but also can ensure their safe social distance. In addition, the transportation of emergency supplies and life necessities should be regarded as the top priority during the epidemic, which are planned preferentially and in a timely fashion.

*Alter transportation procedure* The epidemic has disrupted the original travel plans of many passengers. For nonessential travel, most passengers will choose to apply for refunds. In fact, the government also encourages passengers to cancel travel plans and consciously isolate themselves at home. Therefore, corresponding measures have been introduced to facilitate passengers to go through such procedures. Specific measures include changing the original booking rules, without handling fees. For rail transportation, passenger travel needs to be planned in advance, so the time limit for refunds can be extended, and the scale of online ticket purchases needs to be expanded, providing as much convenience as possible. Some train systems have updated the rules for ticket reservations, increased the reservation ratio and reassigned seats to ensure a safe distance between passengers. In addition, to improve the safety of rail transit systems, the counters were closed, and contactless ticket sales were recommended.

*Strengthen cooperation* To improve the service level of rail transit systems, joint efforts of the whole society are necessary, instead of the railway industry alone. The rail transportation departments of countries all over the world are exploring the new cooperation models with other industries. For instance, working with the human resources agency, which will assess the impact of human losses on the transportation system so that production can be returned to normal as soon as possible when the outbreak recedes. The medical department will provide convenience for emergency delivery to infected people and get access to the latest information on the virus in a timely manner. In return, the rail transit industry can also provide exclusive services, such as customized trains and parking lots. Cooperating with the police could place the station under control, ensure the channelization of passengers and maintain the order of transportation, which is a widely used practice in France, Germany and China.

## Application of Countermeasures Under Different Scenarios

Considering the epidemic situations are different around the world, it is unnecessary to adopt the same degree of epidemic prevention measures globally. To this end, appropriate measures can be taken based on the severity of the epidemic and the risk levels of different regions in the local rail transit.

### The Risk Level of Different Administrative Regions

Because the severity of the outbreak is different from region to region, the response strategies should vary according to the different conditions of the administrative regions. This is conducive to management regardless of whether applying existing rules or issuing new policies. Therefore, it is necessary to grade the risk of different administrative regions according to the situation. Taking China as an example, according to the actual situation and development trend of the epidemic, the country is divided into low-risk regions, medium-risk regions and high-risk regions by city district or street unit, which considers the number of new and cumulative confirmed cases and other factors comprehensively. Accordingly, the following definitions are determined for each type of region. The main characteristics of each type of region are shown in Table 2.

**Table 2:** Main characteristic of each type of region

Risk level (region)	The number of cases within 14 days	Example (March 3, 2020)
High risk	> 50 (concentrated outbreak)	Wuhan, Hubei province, China
Medium risk	> 0 and ≤ 50 (no concentrated outbreak)	Jingmen, Hubei province, China
Low risk	0	Nanjing, Jiangsu province, China

*High-risk region* There are more than 50 cumulative cases that occurred within 14 days and concentrated outbreak occurred.

*Medium-risk region* There are new confirmed cases within 14 days, the cumulative number of confirmed cases does not exceed 50, or the cumulative number of confirmed cases exceeds 50 cases, and no concentrated outbreak occurred within 14 days.

*Low-risk region* There are no confirmed cases or no new confirmed cases for 14 consecutive days.

### The Risk Level of Different Public Spaces in Rail Transit Systems

Because facilities are used with different frequency, the contact frequencies of passengers are different, and the risk levels of these public spaces are different too. The facilities used frequently are the vulnerable spots of the system, which should be focused on to prevent the spread of the virus. As shown in Table 3, based on the frequency of facility use, public spaces in the rail transit system should be classified by different levels of risk. Specifically, the turnstiles, escalators and security check facilities are used by almost every passenger, and the toilet areas are infested with bacteria. These facilities and spaces have a high frequency of usage, are very necessary for most passengers and should be classified as high-risk public spaces. The ticket windows, seats at the waiting rooms, vending machines and convenience stores are usually used by most passengers but can be avoided if they want. These areas can be classified as medium-risk public spaces. The telephone booths, gift stores and inquiry desks are seldom visited by passengers and can be classified as low-risk public spaces. Correspondingly, the preventive measures taken against the epidemic should also be different for public spaces with different risk levels, such as the disinfection frequency.

**Table 3:** Classification of risk levels in public spaces of rail transit systems

Risk level (public spaces)	Characteristic	Typical areas
High risk	Frequently used and bacteria-infested	Turnstiles, escalators, security check facilities, ticket gates, toilets
Medium risk	Nonessential use	Ticket windows, seats, vending machines, stores
Low risk	Rarely used	Telephone booths, stores, inquiry desks

### Matching of Different Measures to Risk Levels

Generally speaking, for public spaces where low-risk regions are located, it is sufficient to adopt basic prevention and control measures. However, with an increased risk level, countermeasures need to be improved and upgraded to guarantee the safety standards. The specific measures that should be taken in regions with different risk levels are shown in Table 4.

**Table 4:** Measures in regions with different risk levels

Risk level	Measures
Low risk	Personal protection; take temperature; offer health necessities; increase relief inventory; enhance air circulation (low); increase disinfection frequency (low)
Medium risk	Low-risk space measures; loading rate control (weak); adjust catering service; improve waste disposal process; provide travel advice; strengthen staff protection; enhance air circulation (medium); increase disinfection frequency (medium); alter transportation procedures
High risk	Low-risk space measures; medium-risk space measures; loading rate control (strong); close public places; encourage remote communication; advise staff to stay at home in spare time; restrict entry for regions; enhance air circulation (low); increase disinfection frequency (high); adjust operation plans

For rail transit in low-risk regions, the daily prevention measures that can be taken are as follows. Most importantly, reserve necessary anti-epidemic materials, while ensuring the frequency of disinfection. Setting emergency handling areas will speed up the reaction to some accidents. To monitor the health of the staff and passengers, temperature taking cannot be omitted. Indirect contact is recommended, and all people should be urged to wear masks, especially in indoor public places. Ventilation systems ought to be ensured within the normal operation. Moreover, it is necessary to increase the ventilation volume and control the loading rate of the train at a reasonable level as mentioned above.

For rail transit in medium-risk regions, the virus may exist in the public space because the cases are discovered in a short period of time. Therefore, measures should be added and upgraded. Specifically, both the travelers' density in public spaces and the loading rate are controlled. The catering service on trains and stations is adjusted as carry-out instead of eat-in. To protect the internal employees from the epidemic, the protection methods for staff are improved, such as setting up separate passages, elevators and toilets for employees. Furthermore, the ventilation volume and the frequency of disinfection should be increased to some extent.

If the rail transit system is exposed to a higher risk of the epidemic, the corresponding measures are more draconian. Taking the rail transit system in China as an example, the station congestion and train load rate in medium-risk regions should be less than 70%, while for high-risk regions, it should become 50%. The carriage in medium-risk regions should be disinfected every 6 hours and the high-risk regions cannot exceed 4 hours. For some specified spaces often touched by passengers, the frequency of disinfection must be increased, which includes ticketing equipment, security equipment, seats, armrests, etc. The frequency of disinfection is every 4 hours in medium-risk regions and every 1 hour in high-risk regions [17]. If the epidemic is severe, there is a basis for canceling some of the high-risk services, like catering and sales. If there is a large-scale outbreak of the epidemic, shutting down the entire system is also a feasible option, and thus, remote communication might be encouraged.

However, regardless of the risk, during the epidemic and consequent disruption of the normal order of society, suspected cases need to be handled properly, and the timely dissemination of information must be ensured.

## Evaluation of the Countermeasures

To analyze the effectiveness and acceptability of the researched countermeasures, a set of evaluation indicators were proposed, which can be used to compare the advantages and disadvantages of the countermeasures. The applicable scenarios of different measures should also be discussed to provide suggestions for the epidemic prevention of the rail transit system in various countries during the epidemic period.

### Determination of Evaluation Architecture

From the above classification of different countermeasures, the means of implementing them include people, equipment and information. To evaluate the consequences of these methods, the evaluation architecture will be established as three aspects, which are expounded as follows.

#### Evaluation Indicators from a Human Perspective

The management of passengers and staff can reflect the consequence of implementing the policy through a series of phenomena. Within a certain range of expenditure, adopting safe, valid and convenient measures can improve the operation efficiency of rail transit. Therefore, the evaluation indicators focus on the epidemic prevention, handling of suspected cases and staff control in this part. The six indicators* effectiveness*, *economic efficiency*, *acceptability*, *privacy*, *comfortableness* and *convenience* are proposed, and their concrete meaning will be discussed in detail as follows:

*Effectiveness* Whether the epidemic prevention measures can prevent the spread in the implementation process is one of the most important indicators in the evaluation architecture. If these measures can prevent the spread of the epidemic at the source, they will play a crucial role in the prevention of the epidemic. In this indicator, the control of external passengers should be considered, as well as the management of internal staff. Specifically, this indicator is used to evaluate the effectiveness of controls on passengers and the effectiveness of internal management measures. It can be regarded that whether the measure taken can maintain a safe social distance for passengers and staff, whether the provided services can ensure the safety of passengers and staff to avoid being infected, whether the measures for handling suspected COVID-19 cases can reduce the release and diffusion of the virus, and whether the internal management measures can prevent virus transmission between employees.

*Economic efficiency *It is necessary to consider the cost of these measures. In the special period, some measures regardless of the cost have been implemented in some countries, especially where the railway sector is controlled by the government, but it has always been an important consideration for some countries with private railway companies [18]. The economic efficiency includes not only the cost of hiring more people and using more equipment, but also the loss of benefits due to the restrictive measure. The economic efficiency of different measures depends on the actual cost incurred by the railway companies. This indicator is not a specific and accurate objective implement cost, but a subjective judgement from the respondents, which reflects the economic tendency for different measures.

*Acceptability* For passengers and staff, the epidemic prevention measures should be acceptable. Excessive measures may cause panic among the public, so the measures should be reasonable and easy to be accepted by the public.

*Convenience* Affected by the epidemic, many measures have been added to the daily management. These measures will have an impact on the convenience of passengers’ travel, and some are severe, and others are weak. As convenience will affect passengers’ satisfaction when traveling [19], it should be taken into account, which is mainly aimed at the measures related to the passengers. Moreover, whether the measures increase the work load of employees should also be considered.

*Privacy* The suspected cases are required to be isolated and the public notified when they travel by rail transit, which may involve the privacy of the passengers or staff. Excessive measures may lead to an invasion of the privacy of passengers or staff. Even in a period of the outbreak, people hope their privacy will be protected as much as possible [20]. Therefore, privacy is one of the evaluation indicators.

*Comfortableness *The comfortableness or service level has always been one of the most important evaluation indicators in the rail transit system. Some epidemic prevention measures may bring better travel experience for passengers, such as space between seats, which may provide more space for passengers. On the contrary, some measures may affect the comfort of passengers, such as taking their temperatures frequently, wearing masks and so on, especially when passengers are not willing to follow the advice. Therefore, although it is a special time, the comfort of passengers should be considered. This will affect the happiness of passengers when taking public transportation [21].

#### Evaluation Indicators from Equipment and Operation Perspective 

The evaluation of the equipment and operation management should focus on the mitigation of virus transmission after the implementation of the measures. Meanwhile, the feasibility of the measures and their economic efficiency, as well as the impact on the passengers’ travel and their own operations, should also be considered. Therefore, six indicators are proposed for equipment and operation management measures. They include the above four indicators (*effectiveness*, *economic efficiency*, *acceptability* and *convenience*) that have been introduced and two new indicators (*operability*, *disturbance*) that will be discussed as follows.

*Operability* The epidemic prevention measures need to be both effective and operational. The indicator is used to evaluate the difficulty in implementing the measures. The measures that are easy to implement are more conducive to preventing and controlling the epidemic and may depend on whether additional equipment and training are required, whether the measures require additional staff and whether there are professional requirements for these staff.

*Disturbance* Under the daily operation plan, the additional epidemic prevention measures may disturb the implementation of the plan. Therefore, the impact of the internal measures on the equipment management and operation plan should be considered in the evaluation architecture.

#### Evaluation Indicators from Information Perspective

Due to the particularity of information dissemination, the evaluation of that will be discussed individually from other points of view, such as the influence, scope, recognition and timeliness. Specifically, three new indicators (*timeliness*, *pervasiveness*, *validity*) will be discussed as follows. In addition, the proposed indicators like *economic efficiency*, *acceptability* and *disturbance* should also be considered to evaluate the measures of information dissemination.

*Timeliness* The timeliness of information is one of the most important evaluation indicators for information dissemination. It indicates whether the message can be timely updated and published. The real-time updates of information can greatly support when travelers making travel decisions.

*Pervasiveness* The extent to which information spreads is also worth discussing. The more widely the information is spread, the more people are likely to receive it and the better the prevention will be.

*Validity* The influence of information on passengers reflects the behavior of passengers after obtaining the information. If the information is valid and the measures are feasible, the passenger will accept the advice of the information. Otherwise, the passenger will continue to follow his/her own habits.

### A Social Survey to Evaluate the Countermeasures in Rail Transit

Based on the classification in Sect. 3, the countermeasures were evaluated by the above proposed indicators. A social survey was carried out to collect public opinions on epidemic prevention countermeasures in rail transit systems. A questionnaire including the above countermeasures and indicators was made for respondents to evaluate. Based on the Likert scale, which is used in the field of psychology commonly, the questionnaire was designed and distributed in the survey (as shown in Appendix 1). Each scale corresponds to a measure of epidemic prevention and control. And each Likert option reflects an evaluation indicator under this measure [22]. The respondents evaluated each method under different indicators by scoring 1 to 6 based on their own views of COVID-19 transmission and railway response. The score of 6 means that the method did pretty well on this indicator, in which the result tends to be worse with the score decreasing until the score equals to 1, which indicates that it has a negative effect under this indicator. In this way, respondents could express their personal views on different measures in the form of scores. This accomplished the purpose of evaluating a certain measure from multiple evaluation indicators comprehensively. Therefore, the scores given by each respondent reflect evaluation results of different measures.

In addition, the questionnaire also collected basic information of the respondents, including age, profession and awareness of epidemic prevention and control in rail transit, which was used to obtain and analyze the differentiated distribution of the respondents. At the same time, the questionnaire was distributed through the Internet, which expanded the scope of dissemination. Based on the respondents’ work and their travel records during the epidemic, the survey results were selected in advance. Only questionnaires indicating that their work was related to rail transit, epidemic prevention or existing travel records were selected. Then, to avoid the disturbance and bias of individual respondents, the survey results were cleaned beforehand. The questionnaires that scored too high (more than 80% of the measures were scored with 6) or too low (more than 80% of the measures were scored with 1) or with too much equilibrium (more than 80% of the measures were scored with the same score) were filtered. Finally, after being selected and cleaned, 94 questionnaires were collected and analyzed within a limited time (2 months). The number of questionnaires may be less at the present stage, but the survey can still reflect the facts to some extent.

**Fig. 3:**
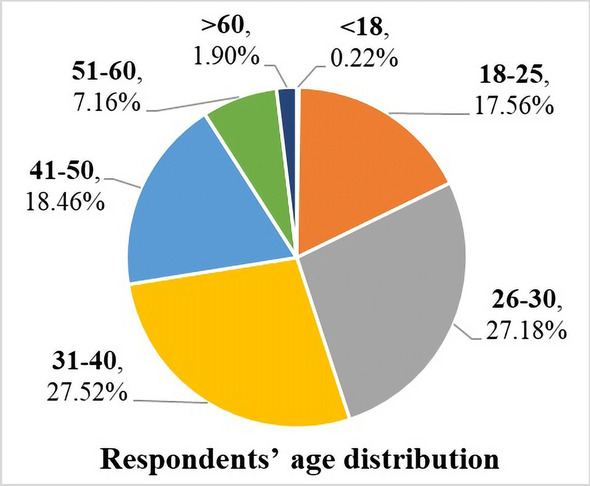
Survey respondents’ age distribution

**Fig. 4:**
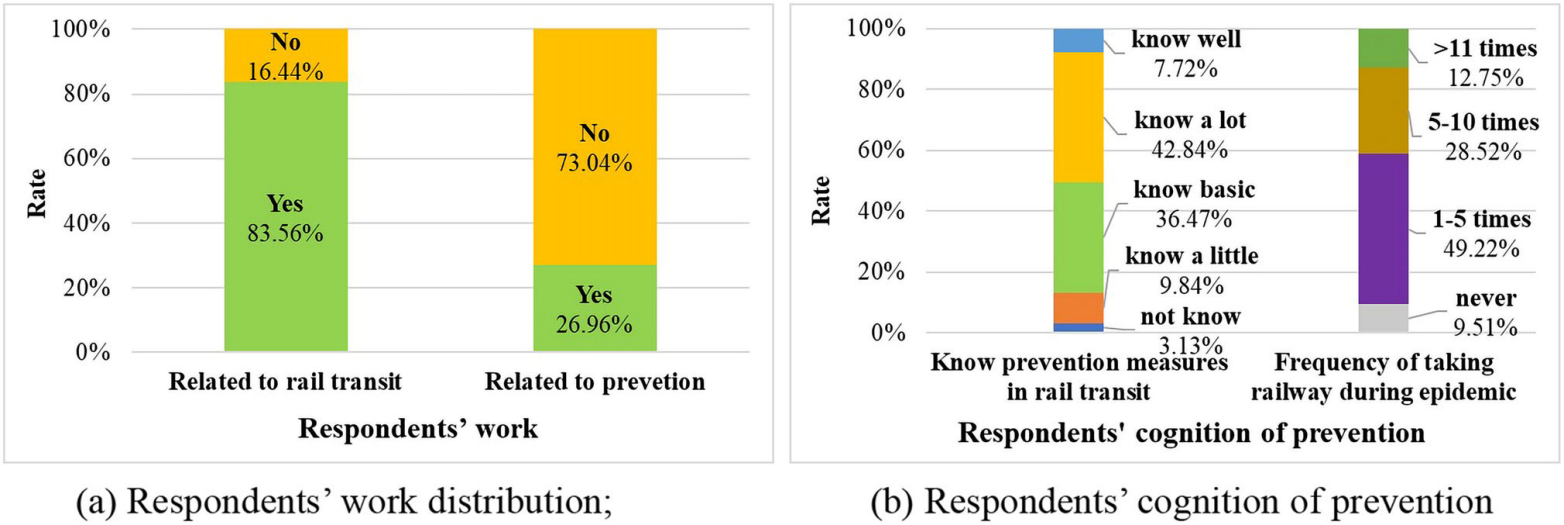
Respondents’ work distribution and awareness of epidemic measures in rail transit during the COVID-19 epidemic

The results showed that the age of most of the respondents who participated in the survey ranged from 18 to 60, which is shown in Fig. 3. As shown in Fig. 4, 83.56% of the respondents participated in work related to rail transit, and 26.96% of them were not engaged in professional medical work. Thus, the knowledge of rail transit may be extensive, but the awareness of epidemic measures may be at a general level. Meanwhile, 90.49% of the respondents have traveled by rail transit during the epidemic, even though the travel frequency was reduced due to the spread of the epidemic. Respondents' work experience and travel experience affect the views on these countermeasures and then affects the survey results. In summary, the questionnaire results that we collected reflect the objectivity of evaluation to a certain extent.

**Fig. 5:**
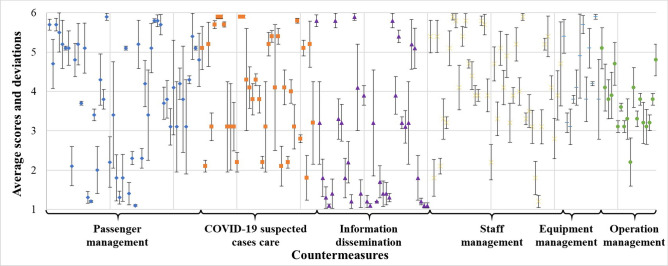
Distribution of scores for different measures

As it is possible to have some interference during the survey, the collected data were preprocessed before the measure analysis. The score distribution of different types of measures under different indicators is shown in Fig. 5. The main consideration is the average score and deviation between different respondents. It can be seen that the deviation scopes corresponding to different countermeasures are different. The smaller scope indicates that the respondents' opinions are relatively uniform, and vice versa.

### Evaluation and Comparison

**Fig. 6:**
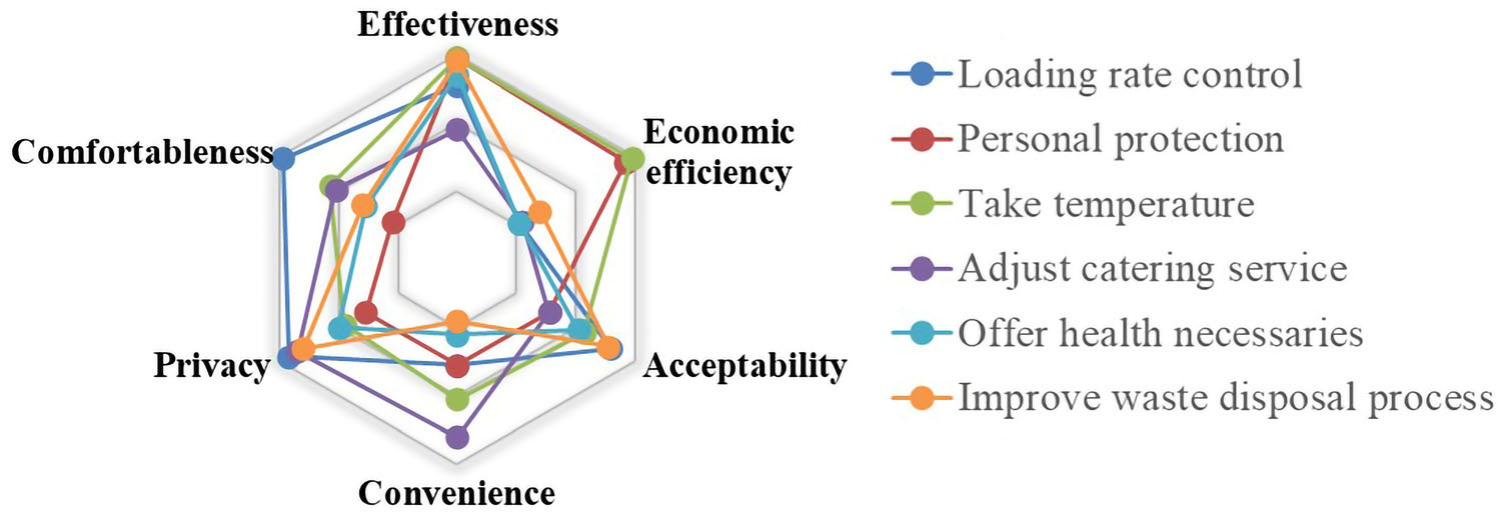
Evaluation results about passenger management on board

**Fig. 7:**
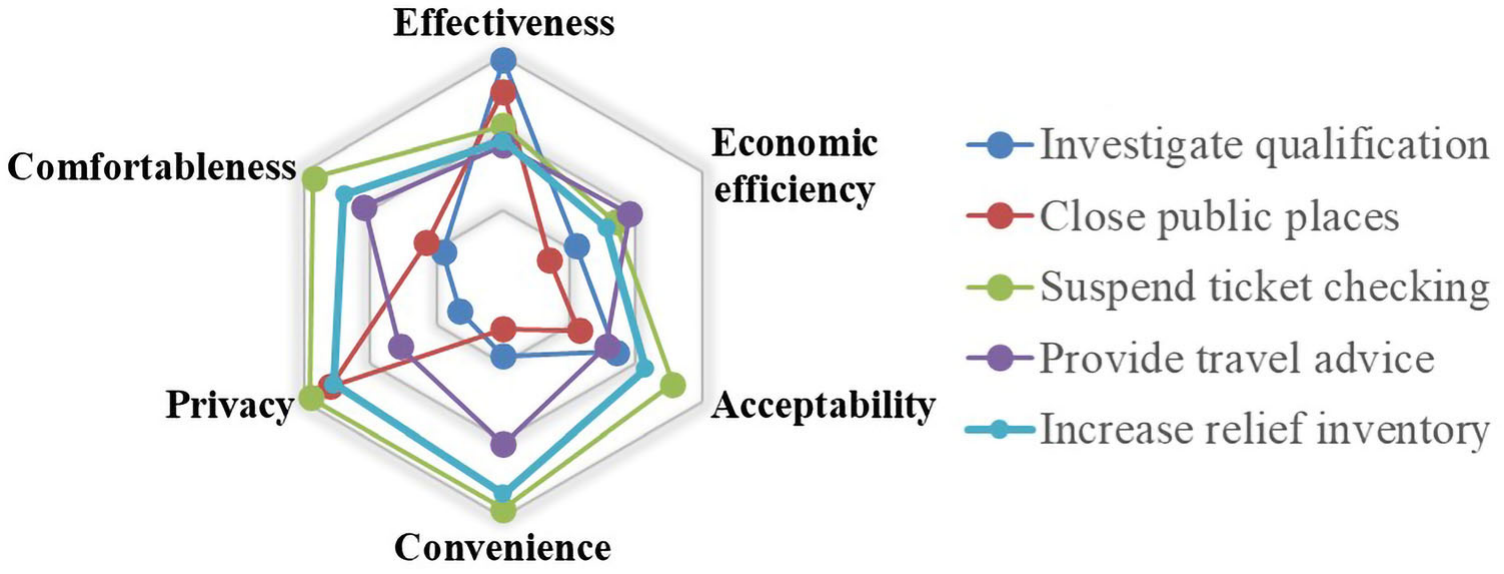
Evaluation results about passenger management at stations

**Fig. 8:**
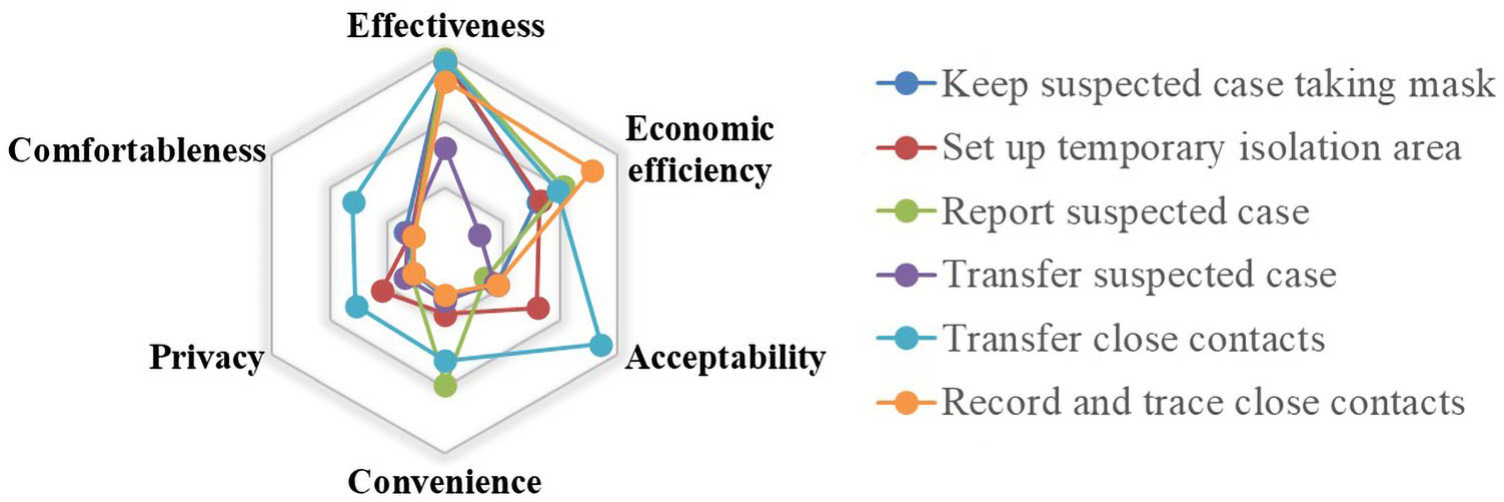
Evaluation results about COVID-19 suspected cases handling

**Fig. 9:**
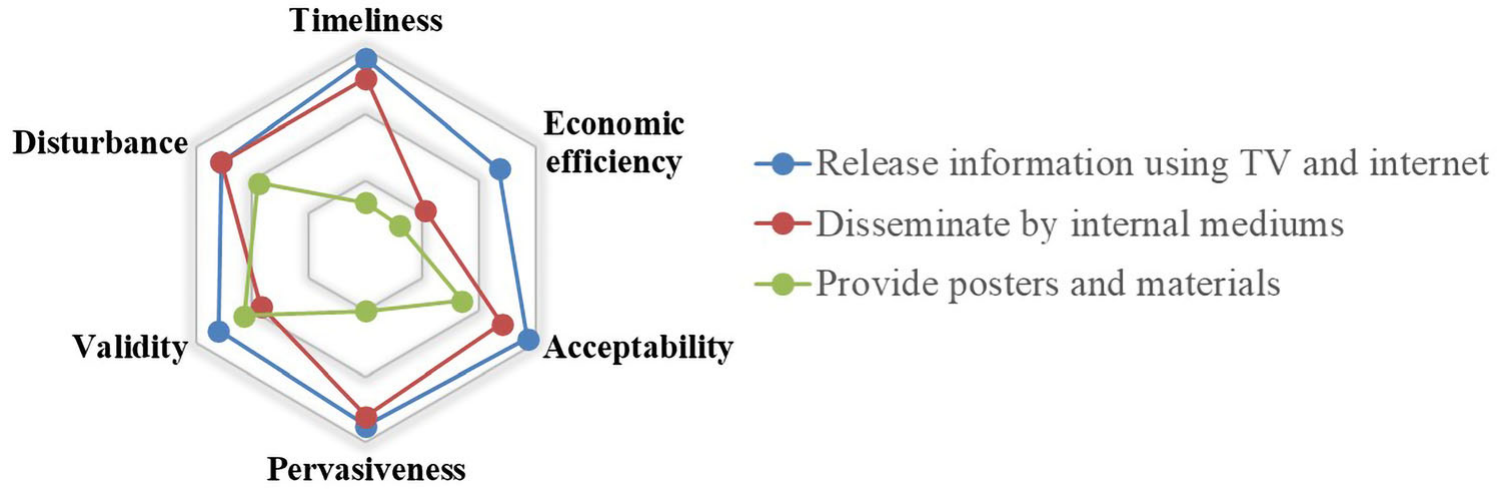
Evaluation results about information dissemination

**Fig. 10:**
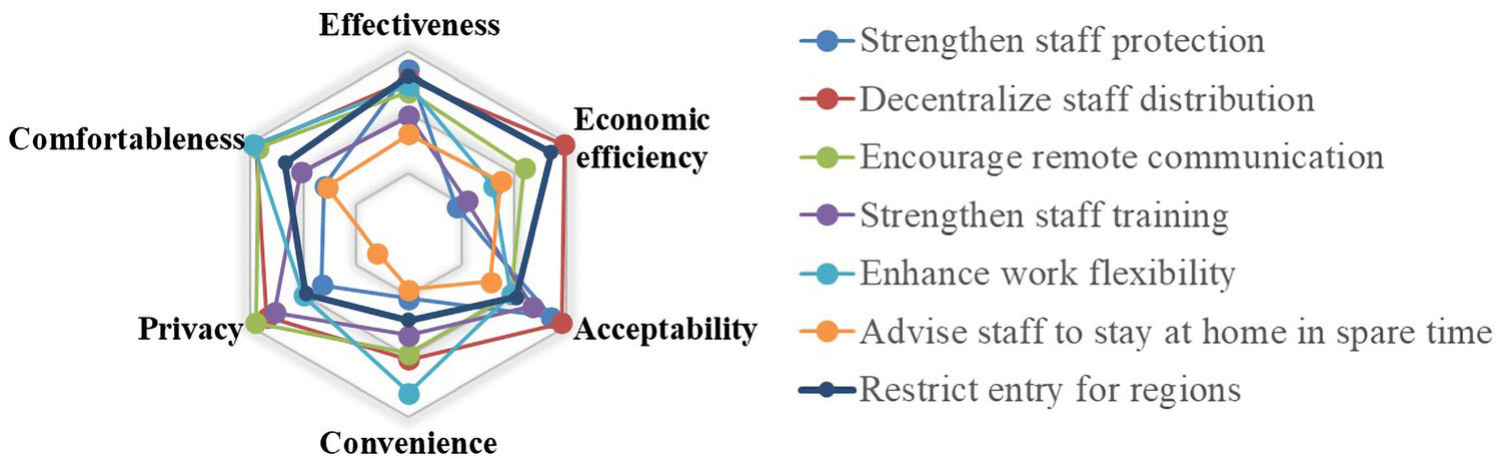
Evaluation results about staff management

**Fig. 11:**
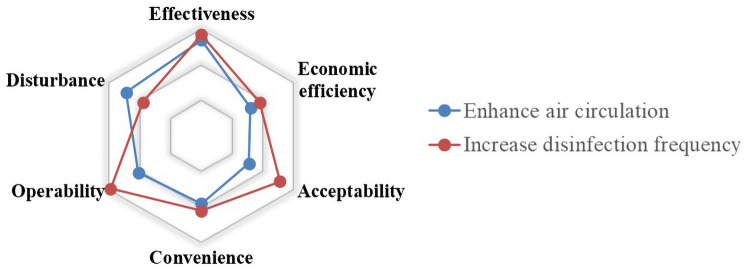
Evaluation results about equipment management

**Fig. 12:**
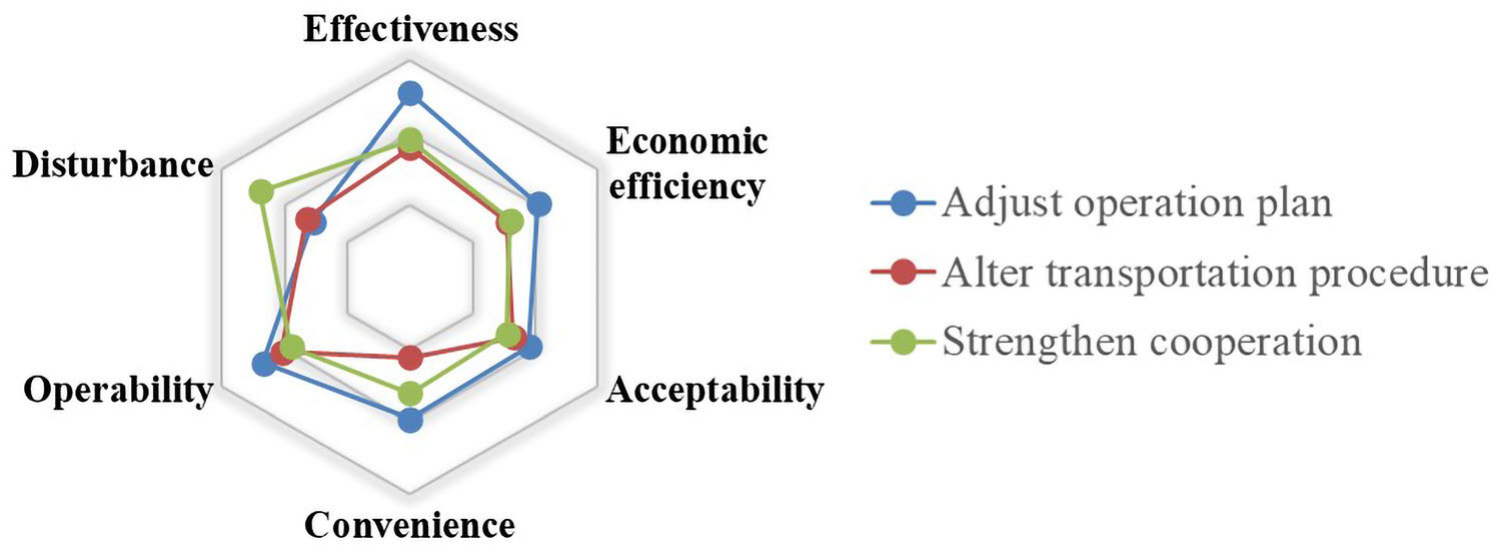
Evaluation results about operation management

With the scores on different measures under different indicators, we have analyzed the data by averaging. The evaluation results are reflected in the radar charts. As shown in Figs. 6, 7, 8, 9, 10, 11 and 12, every closed loop represents a countermeasure, which is formed by the value of each evaluation indicator. If the point is located on the periphery of the radar chart, it indicates that the measure gets a high score and reflects the indicator characteristic well; otherwise, it means the measure gets a low score and has disadvantages under this indicator. In addition, the countermeasures are compared in the same classification, and their advantages and disadvantages are discussed separately.

#### Evaluation of Passenger Management Countermeasures

1. On board

As shown in Fig. 6, the passenger management countermeasures on board are evaluated from six aspects, i.e., effectiveness, economic efficiency, acceptability, convenience, comfortableness, privacy.

In terms of effectiveness, measures such as taking personal protection, taking a temperature and improving disposal waste process may be beneficial. Specifically, taking personal protection is efficient when the passengers are exposed to the public spaces for a long time. The passengers are asked to take masks, use disinfectant and wash hands frequently in public. It should be noticed that some passengers' disposable protective equipment may be reused more than once, which may not isolate the virus and also not be conducive to the guarantee of health. The correct way is to replace the disposable mask at a fixed frequency, and the replacement frequency is implemented in accordance with the regulations of the manufacturer, such as replace it every 6-8 hours or use a mask only within one trip. Randomly taking a temperature on board for passengers can find the people who have a fever and maybe have been infected, which could require timely measures, such as isolation, and ensure the system is in a virus-free (or healthy or clean) state all the time. At the same time, combined with improving disposal waste process, such measures may cut off the possible transmission of the virus inside the train and limit its potential for spread to outside. In comparison, measures such as adjusting catering service may only achieve the goal of protection conditionally. The prohibition of using dining cars may reduce the transmission of the virus effectively, but the measure may be too harsh, especially for the passengers on long-distance trips. Packaging the food for passengers may avoid gathering to eat in the dining carriage, but the respiratory tract of passengers may also not be protected during this activity, and the possibility of infection may increase, because the passengers have to take off their masks while eating.

From the perspective of economic efficiency, taking a temperature and personal protection can be good choices. The former is less costly and more effective than other measures because it can control the virus spread at the source. The latter can reduce the reserves of protective supplies for rail transit, so it reduces economic expenditure. However, the adjustment of catering service, controlling loading rate and offering health necessities should not be recommended when considering the economic efficiency. The rail transit system is no longer to gain profits from the sale of meals and goods during the outbreak when the food service is prohibited. Loading rate control means the wasting of traffic capacity and the increasing cost of rail transportation, which may lead to a negative impact on the profits of operators. In addition, offering health necessities may greatly increase operating costs.

From the perspective of acceptability, improving disposal waste process and controlling loading rate can be promoted. Disposing waste properly only requires necessary protection without excessive technical requirements. Moreover, loading rate control can also be achieved by limiting passenger crowding, entering stations by reservation or restricting the number of tickets on sale. Therefore, the implementation of the above two measures is not difficult. However, measures such as adjusting catering service and requiring personal protection will anger passengers. Meals are inevitable for passengers, especially during long-distance trips. And given that different passengers have different attitudes toward the epidemic, as well as the weather, some passengers may refuse the strict personal prevention for COVID-19.

Considering convenience and comfortableness factors, controlling the loading rate has a better representation. Reducing the number of passengers on board and at stations may provide seats with large intervals and shorten part of the time for buying tickets and taking carriage, thus increasing the comfort of passengers' travel. At the same time, it can also reduce the density of people in public places and increase standing area per capita, making the travel environment more friendly. However, passengers may not be able to board the train successfully due to the limited loading during peak hours. Therefore, it is recommended that the passengers should avoid or reduce travel during the outbreak phase. By contrast, relying on personal protection solely may increase the complexity of passenger travel. If passengers forget to wear masks, they will not be allowed to take the train, which may cause discomfort to some passengers. Requiring passengers to discard their garbage properly in accordance with regulations will also limit the freedom of passengers, which may increase discomfort to the passengers more or less.

From the perspective of privacy, personal information includes identity information, travel data of passengers and work record of staff at the same time. Among the several measures involved, controlling loading rate is the least information gathering from passengers. Other measures may violate the privacy of passengers more or less. In the era of information interconnection, passenger and staff information should be divulged outside of the transportation process as little as possible. The information should be deleted if no infection is found when the incubation period of the virus is over.

In summary, most of the above measures have positive significance in ensuring a virus-free rail transit system. In particular, taking personal protection measures, taking a temperature, and controlling the loading rate, are considered as the most effective measures, but they will also bring inconvenience to passengers or decrease profits for enterprises, respectively.

(2) At station

The countermeasures taken at stations to cope with COVID-19 were evaluated, and the results are shown in Fig. 7.

For effectiveness, measures such as investigating the qualifications of passengers and closing public places are good approaches. For investigating the qualification, taking a temperature is specifically effective. Someone with a fever and a cough will not be allowed to enter the station. This may ensure that most of the passengers of rail transit service are healthy, which can control the virus spread at the source. In this case, it is almost impossible for COVID-19 to spread in a closed carriage. To avoid the special emergency situation (such as the suspected cases were found by temperature screening), the passengers are still not encouraged to take off their masks. The closure of restaurants and shops reduces the possibility of passengers staying and gathering in public space, which can also reduce the spread of the virus effectively.

From the perspective of economic efficiency, providing travel advice has a better effect because it doesn’t require the extra expenditure of operators. However, it may reduce the income of the operation in the long term, because passengers are suggested to have less travel during the outbreak. Meanwhile, it is necessary to obtain their travel behavior when the measure is applied, but protecting passengers’ personal privacy is also important. In comparison, closing public places and suspending ticket checking are not advisable. The closure of restaurants and shops deprives employees of a source of income and reduces the attractiveness of rail transit for passengers at the same time. Suspension of the ticket checking may increase the risk of passengers evading tickets and is not conducive to the revenue accounting of rail transit. Therefore, most of the measures may have a negative effect on the economic efficiency of operators.

In terms of acceptability, convenience and comfortableness, the advantage of suspending ticket checking is obvious, and the disadvantages of closing public places is prominent. Ticket checking cancellation reduces the crowd gathering and contact, improving passengers' passing efficiency, which is acceptable for passengers. It is more inconvenient for passengers to wait for the train without the public services such as stores and restaurants. The service of rail transit is reduced, which may affect the satisfaction of passengers in travel. They have to plan their time before travel to reduce their dwelling time in public areas.

Overall, investigating passenger qualifications before they enter the station is the most effective of these methods, but it is difficult to be accepted by passengers and may make passengers feel encumbered. The measure of suspending ticket checking is the most acceptable measure for passengers, but the effectiveness and economic efficiency of the measure should be further strengthened by reasonable guidance and supervision.

#### Evaluation of Suspected COVID-19 Case Care Countermeasures

The countermeasures that respond to the suspected COVID-19 cases were evaluated, and the results are shown in Fig. 8.

From the perspective of effectiveness, it is known that requiring suspected cases to wear masks and setting up temporary isolation areas are valid measures. Confirmed and suspected cases have already exhibited symptoms such as fever and cough and need to be isolated immediately. To prevent the spread of the virus, requiring all cases and close contacts to wear masks is one of the most effective and safest measures, thus it is mandatory. However, the process of transferring suspected cases is dangerous, because the virus may spread if it is leaked. Therefore, the effectiveness of transferring suspected cases is not obvious.

When evaluated by economic efficiency, recording and tracing close contacts works well. The tracing measures are taken in time before the close contacts leave the rail transit system, in which the close contacts can be recorded quickly. The large-scale personnel inspection that prevents viral spread through close contact can be avoided in the future, which may consume a lot of material and financial resources. Transferring suspected cases is not a money-saving method. When the suspected cases are transferred, the staff must be fully protected by protective clothing and prepare a specific passage for transferal, which may increase the cost of operation.

Most of the measures did not perform well in the indicators of comfortableness, convenience, privacy and acceptability. In the face of COVID-19 cases, the primary task is to protect public health by cutting off the virus transmission route, so the comfort and convenience of passengers may no longer be the first consideration. Meanwhile, it is indispensable to record and trace the information of close contacts, which will involve a large amount of personal privacy, such as name and address. Personal privacy may also be invaded, which is difficult for passengers to accept. Therefore, measures adopted for the suspected cases should pay more attention to the feelings of passengers in the future. Rail transit operators shall protect the personal privacy during the information recording and reporting.

In general, most of the measures may affect the travel experience of passengers a greater or lesser extent, especially in the aspect of convenience, comfortableness and privacy. However, those measures are effective in preventing the spread of COVID-19, and the extra cost should also be invested more or less.

#### Evaluation of Information Dissemination Countermeasures

The three countermeasure of information dissemination, i.e. releasing information using TV and the Internet,  disseminating by internal mediums, providing posters and materials were evaluated, and the results have obvious characteristics, which are shown in the Fig. 9.

From the perspective of pervasiveness, timeliness, acceptability and economic efficiency, the effect of TV and the Internet is better than the other ways from almost all aspects. Nowadays, with the rapid development of technology, the time of releasing epidemic prevention information through TV or the Internet is shorter, and the scope of audiences is wider. Moreover, people can access the news anytime and anywhere, so the effect is better under different evaluation dimensions. In contrast, presswork such as posters or other materials is not effective for efficient information transmission, because the information that it carries is limited. At the same time, the production process needs to consume a certain amount of time, and the rail transit company needs to factor in the design and printing costs. Since it can only be delivered manually, most passengers only can obtain the materials when traveling by rail. But some passengers lack patience to read the information word by word due to their limited travel time, which also reduces the transmission efficiency.

In terms of validity and disturbance, the difference between the three measures is narrow. Compared with other indicators, the implementation effect of internal mediums, posters and materials has been improved, mainly because they are generally set in eye-catching positions. The two indicators mainly describe the impact result of information on passenger travel, which has little relationship with how the information spreads in which way. As a public health emergency of international concern, COVID-19 inevitably interferes with daily life, and passengers will take effective measures to respond for their own lives naturally.

In summary, the release of information by TV and the Internet is one of the most strongly recommended measures for information dissemination, which not only can spread the information rapidly, acceptably and extensively, but can also save the publicity cost. However, a notable concern is that the Internet and TV may not be widely used in some underdeveloped countries, so that measure is only suitable for the countries with mature network and communication systems.

#### Evaluation of Staff Management Countermeasures

Figure 10 shows the evaluation results of the countermeasures about staff management.

From the perspective of effectiveness and acceptability, both strengthening staff protection and decentralizing staff distribution are worthy of reference. Strengthening protection can be required as a compulsory rule for the staff, because they are managed by the enterprise uniformly. It can be regarded as their responsibility for their own safety and that of passengers. Decentralizing the distribution of staff is a feasible and effective measure to carry out. It is similar to the loading rate control in passenger management, but the scenario is changed from the carriage to the office. Advising staff to stay at home in spare time is not a good choice in this view. Considering staff may need to deal with personal affairs in their free time, going outside is largely inevitable.

From the perspective of comfortableness and privacy, decentralizing staff distribution and encouraging remote communication can be further promoted. However, the lack of face-to-face work communication will cause obstacles to the connection of some work, especially for the transmission and filling of some documents with paper only. Remote communication is the most important way during the outbreak. Distributing staff will provide them with more personal space, and the working environment will become better. In contrast, the effect of advising staff to stay at home in spare time is not satisfactory, because it can restrict their freedom in their daily lives and inevitably make them feel uncomfortable. To achieve this measure, staff may be forced to disclose some private information. Such measures should be avoided as far as possible unless the condition is very serious and must be done.

Under the indicator of economic efficiency, decentralizing staff distribution and restricting entry for regions are better. The two measures only change the work scheduling and will not consume excess manpower and material resources. On the contrary, strengthening staff protection requires anti-epidemic supplies. Moreover, if carrying out training for staff, they have to take extra time to gain the necessary knowledge, and the company also needs to hire professionals to teach. Considering that different employees have different learning abilities, different employees in various positions should receive various types of training.

Regarding the convenience of work, enhancing work flexibility reflects this very well. This measure can present the humanity of the enterprise and improve the company's care for staff, especially for some people who are in poor physical condition, which significantly improves the comfort and convenience of work. Strengthening staff protection and advising staff to stay at home in their spare time are not so effective in this way. The main reason is that the epidemic will not end within a short period of time, and daily working hours are long. It is difficult for staff to work with protection and to stay at home during their spare time for a long time.

From the above analysis, it can be seen that decentralizing staff distribution has the best effect under multiple indicators, but its shortcoming lies in the inconvenience caused by working handover. Meanwhile, protection training and staff protection may be necessary for staff during the outbreak, which can help staff learn how to protect both the passengers and themselves, even though it may cost some extra manpower and material resources.

#### Evaluation of Equipment Management Countermeasures

As shown in Fig. 11, the results for the evaluation of the countermeasures for the equipment management are discussed.

Considering effectiveness and convenience, the two measures can reduce the possibility of virus survival in the rail transit system. At the same time, they both require specialized staff to perform this work, which may increase the burden on the staff.

From the view of economic efficiency, the enterprises need to purchase special disinfection supplies and assign specific staff to perform the disinfection work, which requires funding. However, improving air circulation may be more costly, because air filter elements need to be replaced frequently, and the ventilation equipment inside the train and the station need to be reformed to enhance air circulation.

As for acceptability and operability, due to the technical requirements for improving air circulation, it can be carried out only when the company has corresponding technical staff. Therefore, the acceptability and operability of increasing disinfection frequency are higher than that of enhancing air circulation.

In summary, the performance of disinfection measures is better than that of ventilation measures for most aspects, except the disturbance of operations because some equipment cannot be used during disinfection, and the occupation of equipment requires a corresponding scheduling plan, which is also inconvenient for staff to schedule. However, ventilation and disinfection are both important components regardless of whether at the station or on the carriage. As an emergency public health incident, the COVID-19 epidemic is characterized by urgency, complexity, uncertainty and hazard. Although the measures may consume a certain cost, they are effective at protecting the system and preventing viral spread, which is critical during the outbreak.

#### Evaluation of Operation Management Countermeasures

The operation management countermeasures were evaluated, and the results are shown in Fig. 12.

In the process of operation management, the disturbance of operation and the convenience of scheduling are important parts of the evaluation. Strengthening cooperation may cause minimal disturbance to that. The positive significance of cooperation is that it can invite more professionals such as doctors and police to enhance the internal ability to prevent COVID-19. Therefore, the disturbance may be better than the other two measures. When refunding or changing the tickets, it is inconvenient for passengers and requires extra time. Especially in countries where Internet service is not widespread yet, people can only go to the manual window to complete the change.

In the four aspects of effectiveness, economic efficiency, acceptability and operability, the effects of the three measures are not much different, and indicators of the three are not obvious. It is necessary to pay attention to prevent the spread of the virus caused by crowd gathering when altering transportation procedures with a high density of passengers. The measures taken under abnormal circumstances may affect the operating revenue of rail transit. Considering the barriers between different industries and the constraints of the social environment, cooperation between different professions may be difficult during implementation.

In summary, the effect of adjusting operation plans is better because it can directly control the transit supply. The control of operating frequency may have a great effect on preventing the spread of the virus. During the outbreak, the operating frequency is decreased and the passengers are required to reduce their unnecessary trips. During the recovery, the operating frequency is increased to reduce the density of people in the system. The passengers’ trip has been impacted by the COVID-19 epidemic, so has the transportation schedule. During the epidemic, public transportation needs to provide a safe environment for citizens. All countries need to control the scale of transportation according to local conditions. In fact, any measures should be implemented selectively according to the social environment and national macro-policies.

## Conclusion

In this paper, the methods of COVID-19 transmission in rail transit systems were summarized through the analysis of the transmission characteristic of the virus. In this way, the vulnerable spots of prevention in the system were determined, and five main external and internal threats were emphasized. Therefore, the countermeasures to deal with the epidemic in rail transit must be discussed.

The countermeasures to reduce the spread of COVID-19 in rail transit systems were separated into two categories: external management and internal management. The former includes passenger management, suspected cases care and information dissemination measures, which increase passenger entry control efforts, emphasize the response for suspected cases and contain the spread from the source. The latter includes staff, equipment and operation management, which involves strict personnel management and facilities disinfection. Based on the above classification, 32 specific countermeasures are introduced and discussed in detail. An evaluation architecture that includes 11 indicators is proposed to evaluate every measure from six aspects and compare it with others in the same category. Based on a social survey, we collected 94 questionnaires that objectively reflect the evaluation of countermeasures. Afterwards, some suggestions are put forward for countries to respond to COVID-19.

In summary, most of the above measures have positive significance in preventing viral spread in rail transit systems, but the extra cost should also be invested more or less. There are some measures that are both efficient and cost-effective, such as taking personal protection, taking a temperature, decentralizing staff distribution, encouraging remote communication and releasing information by TV and the Internet, which should be recommended to the rail transit operators all over the world. Meanwhile, there are some measures more focused on the travel experience of passengers and working experience of staff, such as controlling the loading rate, suspending ticket checking, encouraging remote communication and enhancing work flexibility, which also need to be considered by the employers. However, there are some measures that are very efficient but may severely affect the experience and privacy of travelers and employees, such as investigating qualifications, reporting suspected cases and recording and tracing close contacts. During the outbreak period, the number of infected increases exponentially, and those measures should be considered first, because the primary task of the operators is to ensure the health of passengers. The comfortableness and convenience of passengers may no longer be the first consideration, while those measures should be taken with caution during the recovery.

In the future, more countermeasures that have been adopted to prevent the spread of COVID-19 will be collected from both people and operations. Furthermore, the social survey will be carried out continuously, and we hope to obtain more information from different industries around the world.

A sudden public safety incident is a great test for the public transportation industry. The outbreak is a wake-up call for rail transit operators in the development of emergency planning, response and other aspects. Only by comprehensively considering all kinds of situations that may occur in transit operation, including the spread of the epidemic, can we prevent the situation before it occurs, improving the emergency response capacity and providing guarantees for the normal operation of society.

## Data Availability

All data generated or analyzed during this study are included in this published article, and they are fully available without restriction.

## Appendix 1: Survey Questionnaire of the Epidemic Prevention Measures’ Evaluation in Rail Transit System

The questionnaire distributed in the social survey collected the respondents’ rating for each measure and also obtained their basic information. The ratings were used to evaluate the proposed measures. The respondents' basic information was used to confirm the characteristics of the respondents and determine the validity of the questionnaire. The respondents who know the rail transit system or the epidemic prevention and control measures will be focused upon greatly. Specifically, the questionnaire is as follows:

Evaluation of Epidemic Prevention and Control Countermeasures in Rail Transit System

At early 2020, with the sweeping of the COVID-19 all over the world, most of the industries have been seriously affected. To prevent the spread of the epidemic, rail transit operators in many countries have taken various anti-epidemic measures. Through statistical processing, the main measures are divided into six aspects: passenger management (trains and stations), suspected case disposal, information release, staff management, equipment management and operation management, which are divided again into 32 specific measures. To analyze the effect of the measures and their impact on the passenger and operation departments, the measures were evaluated from 6 aspects respectively. Please take your time to finish the questionnaire that uses the proposed 6 indicators to score the measures. Before evaluating the anti-epidemic measures, the personal information will be collected. The data that we surveyed only used in research and will not be leaked.

## Code Availability

This article does not include any software application or custom code.
